# Supplementary material for: Patterns of Intron Gain and Loss in Fungi
Source: PLoS Biol. 2004 Nov 30;2(12):e422. doi: 10.1371/journal.pbio.0020422 (PMC532390; doi:10.1371/journal.pbio.0020422)
Supplement: Table S1 — Also available at http://genes.mit.edu/NielsenEtAl/. (4.3 MB ZIP). [file pbio.0020422.st001.zip › NielsenEtAl/html/499.html]

AN3604.1.NCU04111.1.MG09209.1.FG01846.1


```
 CLUSTAL W (1.82) Multiple Sequence Alignments - Introns Inserted


Sequence 1: NCU04111.1	571 aa
Sequence 2: MG09209.1	667 aa
Sequence 3: FG01846.1	549 aa
Sequence 4: AN3604.1	799 aa
Alignment Length: 838 aa
Number Identitical Residues: 236 aa
Alignment Score (without introns) 12977


MG09209.1 	-----------------------------~------------------------------
NCU04111.1	-----------------------------~------------------------------
FG01846.1 	-----------------------------~------------------------------
AN3604.1  	MGSTLPYLKTNPQIIFFSDFDGTITIDDT1LDLSRSSQILIKTLCQTDNLGFGQEKRRQL
          	 .:: .  .:...    :. ..: : ..:  . : ::.   .: ..:.. . ....  . 

MG09209.1 	------------~-----------------------------------------------
NCU04111.1	------------~-----------------------------------------------
FG01846.1 	------------~-----------------------------------------------
AN3604.1  	NKDVLENKVTFR2DSFREMLDSVPNAFDDCLEILKKNMRLDPHFKEFYYWAKENNVPIVI
          	...  ... :   .:  .  .: ..: ... .  ...   ..  ..    :.... .   

MG09209.1 	-----MLVASWLFGTAYGAPKDKDIWLWVDKELRSDFFQINICG---------L2TLDYK
NCU04111.1	------------------------------------------------------~-----
FG01846.1 	------------------------------------------------------~-----
AN3604.1  	LSSGMIPVIRTLLETLLGHNIDDHLTIVANDVESRDGRDINTPGGWQIKYHDDT2HFGHD
          	 ::.          :  .   ..      ..    .  . .  .. . .  ..    . .

MG09209.1 	GTLKWTRHDAVCDARWPLDGNAGIRCYVHQHISSVLHLIAKRTSS~YWQYRGARPVPSRS
NCU04111.1	---------------------------------------------~--------------
FG01846.1 	---------------------------------------------~--------------
AN3604.1  	KSLEIKPYAAVPFDERPTLLYAGDGVSDLSAASETDLLFAKAGKD1LITYCEREGMPYTV
          	 : . .   :      .    :.      .  :.     :.  ..           .   

MG09209.1 	IRLP----------IMARRI0VRTAIQVVTAALACFLIVFFLDRNFRVLPNSIHEYMPQH
NCU04111.1	---------------MTRRI0VRNIIQISTAAVITFLLIFFLDRNFRVLPNAIHEYLPTH
FG01846.1 	---------------MTRRI0VRTAAQLAGAALFTFLVIAFLDRNYRVLPNSIHGYMPSH
AN3604.1  	FEDWSSILATTKDILSGRMK0VRAGVQLAIFASFALLLIVTLDNRFRVLPAAIHGHLPSH
          	    ::  :::..    *   **   *:   *   :*::  **..:**** :** ::* *

MG09209.1 	HPGLLVTDITIQKCSSLNPFSSCKLDPEIWHRVEKDLYLGKALASSAWLHIRRQKESELK
NCU04111.1	HAGLVITDITVTTCTSYNPFSSCDLDTSVWHRVDKDLHLGKTLFSHAYLHVSRKREEELK
FG01846.1 	HPGLIVTDVTIVTCSSVNPFSSCELN-KEWHRVDKDLYLGRTWTTSAYLYVMRKHEEDLT
AN3604.1  	YAGFVVTDVTVATCSILNPFSSCKSSAPWWTQVEKDLYLRTGWTSAAYIRFQRKKEEELL
          	:.*:::**:*: .*:  ******. ..  * :*:***:*     : *:: . *::*.:* 

MG09209.1 	GDDKVVIDVTVGRLDPSENGKRGQSG-------EAWESRPGGLWLKRSGKLHAGDSDRAV
NCU04111.1	EGDKVVVDVTVGRLDPSVADAEKAAAKGLDIKNEKWESRPAGLWVKRSAKRGASDSKNAV
FG01846.1 	EEDRVVVDVSVGRLQPQPKGET----------DDAWESRSSGIWIKRSAKRKASDSKQAV
AN3604.1  	PADKVVIDLKISRLAPEVQDDPKEDK-------ADWEQRPGGIWLKRTAKRHASDSHSAI
          	  *:**:*:.:.** *.  .               **.*..*:*:**:.*  *.**. *:

MG09209.1 	TTVDLLFGDDAVEVRPGWEIVGTPLILDASSSIPSAHLTVRRGSHPAEIPKPKLRVQENG
NCU04111.1	TGIDVLFGDDAVEAREAWSMTGTPLLLDVGRSVPSAHLTVRRGVEHPPV-KPVLRIRDNG
FG01846.1 	TDIDVLFGDDAVEARDGYALTGTPLLLNTGGNLLCVQLTVRRG-PVREAKKPTPRIRNDG
AN3604.1  	TSVDVLFGADAVDPRPGWEVKDTPILLDSRTESLEARITVRRG-DPLKIKKPVPRINENG
          	* :*:*** ***: * .: : .**::*:   .   .::*****       **  *:.::*

MG09209.1 	NFKVMQVADLHLSTGVGHCRDAVPDSYH-GGRCEADPRTLEFVSKMLDDEKPDLVVLSGD
NCU04111.1	KFKIVQLADLHLSTGVGACRDALPE----GQKCEADPRTLDFVTKILEEEKPDLVVLSGD
FG01846.1 	RYKIMQIGDLHLSTGVGACREAVPDSYN-GGKCEADPRTLDFVNRVLDDEKPDLVVLSGD
AN3604.1  	RFKIMQLADLHLSTGLGHCRDPVPPELIPGQGCEADPRTLDFIERLLDEEQPDLVILSGD
          	.:*::*:.*******:* **:.:* .  .*  ********:*: ::*::*:****:****

MG09209.1 	QVNGGTAPDVQS0AIFKYAALLIQRKIPYVSIFGNHDDEKGVMSRASQMAIIEALPYSLS
NCU04111.1	QVNGETAPDTQT0AIFKYAQILIKHKIPYVSIFGNHDDE-GSMSRAAQMDLIETLPYSLS
FG01846.1 	QVNGDTAPDAPT0AMFKIVSLLIERKIPYAAIFGNHDDE-KTMSREAQMAIMESLPFSLS
AN3604.1  	QVNGETSRDAQS~PLFKSVKLLVDRKIPYAAIFGNHDDE-GNLDRHQSMAILEDLPYSLS
          	**** *: *. : .:** . :*:.:****.:********   :.*  .* ::* **:***

MG09209.1 	TAGPEDVDGVGNYYIEVLGRGMSSHAAITIYMLDTHSYSPNERKYPGYDWLKKSQIDWFQ
NCU04111.1	RAGPESIDGVGNYFIEVLGRGSSSHSALTVYLLDTHAYSPNERKYPGYDWIKPNQIEWFR
FG01846.1 	TAGPADIDGVGNYYVEVLARGKTDHSALTIYLMDTHAYTPDERNFPGYDWVKPNQIEWFK
AN3604.1  	SAGPEDIDGVGNYIVEVLGRGNTDHSALTLYLLDSHSYSPDERQFRGYDWIKPNQIRWFK
          	 *** .:****** :***.** :.*:*:*:*::*:*:*:*:**:: ****:* .** **:

MG09209.1 	KTAQGLKQKHKAYTHIHLDVSFIHIPLPEYRE-PDQLMVGKYVEPVTAPVFNSGFRDALV
NCU04111.1	QTAQGLKKKHREYTHVHMDVAFIHIPLPEYQNGRNLTLVTSWKEPTTAPTFNSGFHDALV
FG01846.1 	KTAAGLKKNHNEYTGRHMDIAFIHIPLTEYAD-PALPRVGEWKEGVTAPVYNSGFRDALV
AN3604.1  	TTAQGLKAKHQQYAYMHMNMAFIHIPLPEFAQ-RGNYFRGNWSEPSTAPGFNSGFKDALE
          	 ** *** :*. *:  *::::******.*: :        .: *  *** :****:*** 

MG09209.1 	SEGVTLVGCGH2----------------DHVNDYCALSMNEQ----EPKMWMCYAGNVGF
NCU04111.1	EEGVAMVSCGH2SFPKFGSTLTFPSPHSDHVNEYCAVDFKEDG---KPALWMCYAGAAGF
FG01846.1 	EQGIVMVSAGH2----------------DHCNDYCSLSLAGEGETKIPALWMCYAGGSGF
AN3604.1  	EEGILFVGCGH2----------------DHANDYCALSKNEAQ---KPSLWMCYGGGAGF
          	.:*: :*..**                 ** *:**::.         * :****.*  **

MG09209.1 	GGYAGYGGYDRRIRMYEFDMNEGRITTWKRLENAEDKSLLKQRIDEQLAVDAGKAHPPPE
NCU04111.1	GGYAGYGGFHRKIRVFDFDMNEGRINTWKRVEYGED---VDKKIEEVMIVDGGHPVRPMQ
FG01846.1 	GGYAGYGGYHRRVRLFEVDTNEARIKTWKRLESGDT----ASRIDLQIIVDGGKPQ-PIA
AN3604.1  	GGYGGYGGFIRRVRFFDFDMNPGRVVTYKRLEYGNT----DARIDEMMIVDGGMVKGPD-
          	***.****: *::*.::.* * .*: *:**:* .:       :*:  : **.*    *  

MG09209.1 	DVNDD
NCU04111.1	D----
FG01846.1 	PVS--
AN3604.1  	-----
          	
```
